# Predictors of Recurrence of Chronic Subdural Hematoma Among Adult Filipinos After Surgery: Developing a Preliminary Model for Prognosis

**DOI:** 10.7759/cureus.82599

**Published:** 2025-04-19

**Authors:** Charlene Mary C Mercado, Jonna Mae D Maala, Maurice V Bayhon, Erickson F Torio, Ibet Marie Y Sih, Roy G Torcuator, Rhoderick M Casis, Manuel M Mariano

**Affiliations:** 1 Neurological Surgery, St. Luke's Medical Center, Quezon City, PHL; 2 Neurological Surgery, Philippine General Hospital, Manila, PHL

**Keywords:** predictive model, prognosis, subdural hematoma, subdural hematoma recurrence, trauma

## Abstract

Objective: The objective of this study is to develop a preliminary model for prediction of chronic subdural hematoma (CSDH) recurrence for adult Filipinos who underwent CSDH surgery.

Methods: This study addresses a retrospective cohort of 71 adult patients who underwent CSDH surgery at a tertiary medical center in the Philippines. The outcome of interest was recurrence of CSDH detected on post-operative scans after discharge. Candidate predictors were chosen a priori based on our institutions experience and available literature. Multivariate logistic regression analysis was performed to determine the final model guided by Akaike Information Criteria (AIC) and receiver operating characteristic (ROC) curve criteria.

Results: A total of 71 adult patients underwent CSDH surgery at our institution. Seven (9.9%) had recurrence of subdural hematomas, where four patients underwent repeat CSDH surgery, and three were managed conservatively. The analysis revealed that bilateral surgery (odds ratio (OR) = 0.06; SE 1.35; 95% confidence interval (CI) of 0.01, 0.78; p = .032), intraoperative tranexamic acid (OR = 0.03; SE 1.68; 95% CI of 0.01, 0.87; p = .042), and length of hospital stay (OR = 1.18; SE 0.08; 95% CI of 1.01, 1.41; p = .032) were independently associated with CSDH recurrence. The final model was significantly better at predicting recurrence compared to baseline probability (Wald χ² (6, N= 71) = 18.427, p = .005). The model had good sensitivity (SE) and specificity in predicting recurrence (area under the curve (AUC) = 0.911; SE 0.054; 95% CI of 0.806 to 1.000; p < .001). A Youden’s index value (J_max_ = .716) corresponds to a predicted probability of 0.171 (SE = 85.7%, Specificity = 85.9%, positive predictive value (PPV) = 40.0%, negative predictive value (NPV) = 98.2%).

Conclusion: The authors have a developed a preliminary model predicting recurrence after surgery for St. Luke’s Medical Center, one of the largest tertiary hospital systems in the Philippines.

## Introduction

Chronic subdural hematoma (CSDH) recurrence rates after surgery vary from 5% to 37% in literature [1,2]. This wide range is heralded by the diversity of the clinical, radiographic, and immediate post-operative characteristics of CSDH patients, as well as the differences in treatment approaches and surgical techniques. There is difficulty in prognosticating the risk of recurrence for a post-operative patient based on literature alone; hence, many authors have published predictive models for CSDH recurrence. However, the beta coefficients, intercepts, and odds ratios (ORs) are often incompletely reported. In addition, model receiver operating characteristic (ROC) curves, C-statistics, validation tests, among others have not been shown and it might be difficult to assume model performance.

The aim of this investigation is to create a preliminary model to estimate the probability of recurrence in a CSDH patient who has undergone surgery at our institution to lay the foundations for the authors to conduct prospective external validation studies to better assess the risk of recurrence for Filipinos. By creating our own predictive model from the analysis of demographic, clinical, radiographic, and surgical parameters, we do not assume that Filipinos with CSDH are the same with other countries. This may highlight differences prevalence in the pathophysiologic mechanisms of CSDH responsible for the current difficulty of predicting risk. In addition, predictive models may highlight the consensus of beliefs among surgeons in institutions and also use variables that are of more practical use in developing nations. In doing so, we can create a more accurate risk profile for our patients and enhance our capability to prognosticate CSDH patients in a developing country.

## Materials and methods

Participants, eligibility, and data source

This is a retrospective cohort study of consecutive adult patients who have undergone surgery for CSDH at two tertiary care centers under one hospital system (St. Luke’s Medical Center, Metro Manila, The Philippines) from June 2015 to June 2020. Data from the Section of Neurosurgery census, the hospital’s electronic medical records (charts, laboratory exams, imaging), and clinic follow-up data within three months from discharge were collected.

Included in the study were adult patients (>18 years) with CSDH who underwent surgery between June 2015 and June 2020. Patients who belonged to the pediatric population, had no evidence of CSDH on neuroimaging, did not undergo surgery for CSDH, or had previous surgery for a CSDH and was reoperated on during the same admission were excluded.

We extracted routine data from the electronic medical records, including patient baseline characteristics, clinical presentation, radiographic imaging, disease course, surgery, and follow-up. All patient data were entered anonymously into a web-based electronic form, only using a study identifier. The coding manual and key linking patient information was saved in a local protected file that was not available to the researchers performing data analysis. This retrospective analysis of clinically acquired data was approved by the institutional review board of the hospital (Institutional Ethics Review Number SL-20290), and informed consent was waived due to the retrospective nature of the study.

Surgery for CSDH

Surgical evacuation is the mainstay in the management of CSDH [3,4]. Three techniques are being practiced in our institution: (1) burr-hole craniectomy; (2) mini-craniotomy; and (3) BurrShield-a burr-hole craniectomy followed by placement of a fenestrated titanium implant over the bony defect. The technique employed depends on the neurosurgeon’s preference. Regardless of technique, surgery results to rapid improvement of patient’s symptoms [4].

Surgery was carried out for the following indications: (1) Unilateral or bilateral CSDH with maximum thickness of ≥1 cm and/or midline shift of ≥0.5 cm; (2) CSDH of any thickness or midline shift with neurologic deterioration.

Surgery was not carried out if the maximum CSDH thickness was ≤1cm with a midline shift of ≤ 0.5cm in patients with no neurologic deterioration, and if there were major contraindications to surgery.

Clinical endpoints and outcomes

The outcome of interest in this study is the presence of recurrence of CSDH. Recurrence is defined as the radiographic evidence of a reappearance of a hematoma or an increase in the thickness of CSDH on the same side of surgery with clinical signs and symptoms requiring increased observation or medical or surgical therapy within three months from initial surgery for CSDH. Recurrence was considered a dichotomous variable.

Candidate predictors of recurrence

The potential predictors of CSDH recurrence set a priori by the authors include: history of anticoagulants or antiplatelets, sum of subdural thickness (cm), midline shift (cm), Nakaguchi radiographic characteristics (layering, trabeculations, or septations; laminar, mixed density; homogenous), bilateral surgery, surgical technique (BurrShield, burr-craniectomy, mini-craniotomy), Jackson-Pratt drain (JP) site, operating room time, operating room blood loss, intraoperative tranexamic acid, post-operative residual deficit, and length of hospital stay [5]. These data were collected from patient charts, operative techniques and laboratory results and added to a database.

Sample size estimation

A sample size of 89 was calculated based on parameters from a study by Yamamoto et al. [6]. The sample size was calculated using Power Analysis and Sample Size Software version 8.0 with power and significance set to .80 and <.05, respectively, using a two-tailed test.

Missing data

Missing data patterns were analyzed. Multiple imputation will be done for any missing values deemed missing completely at random or missing at random. Constraints were set based on the minimum and maximum values the variables can achieve. The results were pooled.

Statistical analysis

The patients included in the study were summarized using the predictors of clinical relevance for the authors’ institution using descriptive statistics. Categorical variables were presented as counts and percentages. Continuous variables were described using measures of central tendency depending on the normality of data. Kaplan-Meier survival analysis was used to describe recurrence. Candidate predictor selection was based on clinical knowledge and literature review. Student’s t-test, Mann-Whitney U and a chi-square test were performed on baseline demographics and on candidate predictors for univariate analysis. Backward step-wise multivariable logistic regression was used to build a model. If collinearity was encountered, the predictor with greater clinical relevance was prioritized. Candidate predictors that were not significant on univariate logistic regression but had clinical relevance based on author’s consensus were forced into the model. All tests were two-sided and p values were considered significant when they were less than .05.

The full model was evaluated for its calibration and discrimination factors. An ROC curve to estimate the model’s area under the curve (AUC) and performance. Analysis was performed using SPSS version 26.0 statistical software.

Creation of a predictive model

A predictive model was created from backward step-wise logistic regression analysis using the potential predictors of CSDH recurrence derived from the institutional database. The best fit model was based on clinical utility, goodness of fit, statistical significance, Akaike Information Criteria (AIC), and AUC analysis.

## Results

Baseline characteristics of patients and clinical presentation

A total of 71 patients were analyzed (Table 1). The mean age was 74 years (range 21-94). Female to male ratio was 0.2/1 (15/71). Prior to diagnosis with CSDH, 57 patients (80.3%) had hypertension, and 37 (52.21%) had diabetes mellitus. 24 patients (33%) had a history of antithrombotic intake for the following conditions: coronary artery disease (26.8%), atrial fibrillation (12.7%), and acute ischemic stroke (11.3%). Seven patients (9.9%) underwent hemodialysis for chronic kidney disease. Five patients (7.0%) were diagnosed with dementia.

**Table 1:** Baseline clinical and laboratory characteristics (N = 71)

	Mean ± SD, median (range), Count (%)
Age	74.0 (21 to 94)
Female	15/71 (21.1)
Comorbidities (specific)	
Hypertension	57 (80.3)
Diabetes mellitus	37 (52.1)
Atrial fibrillation	9 (12.7)
Chronic kidney disease or hemodialysis	7 (9.9)
Coronary artery disease	19 (26.8)
Acute ischemic stroke	8 (11.3)
Dementia	5 (7.0)
Comorbidities (number a patient has)	2 (0 to 5)
0	8 (11.3)
1	22 (31.0)
2	21 (29.6)
3	13 (18.3)
4	6 (8.5)
5	1 (1.4)
Clinical	
Pre-operative Glasgow Coma Scale	15 (7 to 15)
Neurologic deficits	48 (67.6)
Motor deficits	46 (64.8)
Decreased sensorium	23 (32.4)
Anisocoria	1 (1.4)
Coagulopathy	31 (43.1)
Anticoagulant or antiplatelet medications	27 (38.0)
Deranged coagulation tests	17 (23.9)

The Glasgow Coma Scale score on admission ranged from 7 to 15. A neurologic deficit was recorded in 48 patients (67.6%). 23 patients (32.4%) presented with decreased sensorium. Only one patient was anisocoric. There was no significant difference in baseline clinical characteristics.

Radiographic characteristics

All patients underwent neuroimaging (CT or magnetic resonance imaging). The findings are summarized in Table 2. The average midline shift was 0.80 cm, while the average total hematoma thickness was 2.50 cm. Classifying hematomas into four types based on the Nakaguchi classification has been used for predicting the risk of post-operative recurrence [6]. Most patients had hematomas that were non-homogenous and had mixed density (59.2%), followed by those with separation (layering) (28.2%), trabeculations (septations) (23.9%), and laminar appearance (15.5%). There was no significant difference among the imaging characteristics.

**Table 2:** Radiographic characteristics (N = 71)

	Mean ± SD, median (range), Count (%)
Midline shift (cm)	0.80 (0 to 1.80)
Bilateral (cm)	0.48 ± 0.42
Unilateral (cm)	0.82 ± 0.57
Total subdural thickness (cm)	2.50 (0.70 to 6.50)
Unilateral	2.23 ± 0.88
Bilateral	3.49 ± 1.34
Age of subdural hematoma	
Early subacute	0 (0)
Late subacute	40 (56.3)
Chronic	69 (97.2)
Chronic with acute components	52 (73.2)
Nakaguchi classification	
Layering	20 (28.2)
Trabeculations or septations	17 (23.9)
Laminar	11 (15.5)
Mixed	42 (59.2)
Homogenous	27 (38.0)

Surgical characteristics

Hematomas were unilateral in 50 patients (70.4%) and bilateral in 21 (29.6%) (Table 3). Among these surgically managed patients, 50 (43.7%) were treated with burr-hole craniectomy, 21 (29.6%) with mini-craniotomy, and 19 (26.8%) with BurrShield. The average operative time and blood loss were 70 minutes and 30 mL, respectively. The insertion of a JP was conducted in all patients, with 47 drains (66.2%) in the subdural space and 24 drains (33.8%) in the subgaleal space. The median time to removal of the JP was 72.56 hours, and at the time of removal, the drain output was 92.23 ± 22.85 mL on average, with either serous (45.1%) or serosanguinous (54.9%) character.

**Table 3:** Surgical characteristics (N = 71) JP: Jackson-Pratt drain

	Mean ± SD, median (range), Count (%)
Laterality	
Bilateral	21 (29.6)
Unilateral	50 (70.4)
Left	25 (50.0)
Right	25 (50.0)
General technique	
Mini-craniotomy	21 (29.6)
Burr-hole craniectomy	50 (43.7)
BurrShield	19 (26.8)
JP location	
Subdural	47 (66.2)
Subgaleal	24 (33.8)
Operative time (minutes)	70 (15 to 300)
Blood loss (mL)	30.0 (5 to 400)
Post-operative	
Glasgow Coma Scale	15 (12 to 15)
Neurologic improvement	71 (100.0)
Residual deficits	6 (8.5)
JP	
Time from surgery to removal (hours)	72.56 (22 to 170)
Quantity 24 hours before removal (mL)	92.23 ± 22.85
Bilateral	98.92 ± 79.91
Unilateral	91.58 ± 88.35
Quality	
Serous	32 (45.1)
Serosanguinous	39 (54.9)
Recurrence	7/71 (9.9)
Mean recurrence (days)	35.45 ± 42.43
Mean follow-up (days)	86.24 ± 109.05

All patients improved neurologically after surgery. Only six patients (6.5%) had residual hemiparesis during the immediate post-operative period. Seven patients (9.9%) experienced CSDH recurrence following initial surgery (Appendix 1). The mean interval to recurrence was 35.45 ± 42.43 days while the mean follow-up time was 86.24 ± 109.05 days.

Univariate analysis

On univariate analysis, total subdural thickness (p = .044) and laterality (p = .034) were significantly different between the groups that did and did not show recurrence (Table 4).

**Table 4:** Univariate analysis of candidate predictors for CSDH recurrence CSDH: Chronic subdural hematoma; OR: Odds ratio; CI:  Confident interval; JP: Jackson-Pratt drain

	β	Wald χ²	df	Crude OR	95% CI for OR	p-value
Clinical						
Anticoagulants or antiplatelets	-0.442	0.24	1	0.643	0.110, 3.766	0.624
Radiographic						
Sum of thickness (cm)	0.616	3.452	1	1.851	0.967, 3.544	0.063
Midline shift (cm)	0.392	0.204	1	1.479	0.271, 8.08	0.651
Nakaguchi						
Layering	1.056	1.485	1	2.875	0.526, 15.712	0.223
Trabeculations or septations	0.634	0.474	1	1.885	0.31, 11.449	0.491
Laminar	1.198	1.605	1	3.312	0.519, 21.131	0.205
Mixed density	0.301	0.111	1	1.351	0.23, 7.946	0.739
Homogenous	-0.301	0.111	1	0.74	0.126, 4.351	0.739
Surgical						
Bilateral surgery (yes)	-1.792	3.852	1	0.167	0.028, 0.997	0.05
Technique						
BurrShield		1.249	2			
Burr-craniectomy	0.054	0.001	1	1.056	0.061, 18.172	0.97
Mini-craniotomy	1.035	0.799	1	2.815	0.291, 27.206	0.371
JP site						
Subgaleal				1		
Subdural	0.821	0.53	1	2.273	0.249, 20.741	0.467
Operative time (minutes)	0.005	0.364	1	1.005	0.989, 1.02	0.546
Operative blood loss (mL)	-0.005	0.23	1	0.995	0.976, 1.015	0.631
Intraoperative tranexamic acid	0.862	1.003	1	2.368	0.438, 12.807	0.317
Post-operative residual deficit	1.099	0.823	1	3	0.279, 32.209	0.364
Length of hospital stay	0.062	2.816	1	1.064	0.99, 1.145	0.093

Multivariate analysis

After eliminating variables that were closely related to others and ruling out variables with collinearity, the analysis revealed that bilateral surgery (OR = 0.06; Sensitivity (SE) 1.35; 95% CI of 0.01, 0.78; p = .032), intraoperative tranexamic acid (OR = 0.03; SE 1.68; 95% CI of 0.01, 0.87; p = .042), and length of hospital stay (OR = 1.18; SE 0.08; 95% CI of 1.01, 1.41; p = .032) were independently associated with CSDH recurrence (Model Wald χ² (6, N = 71) = 18.427 (p = .005). Covariates included in the model include: a history of anticoagulant or antiplatelet medications, JP site (either subdural or subgaleal), and operative blood loss (mL).

Model performance

**Figure 1:**
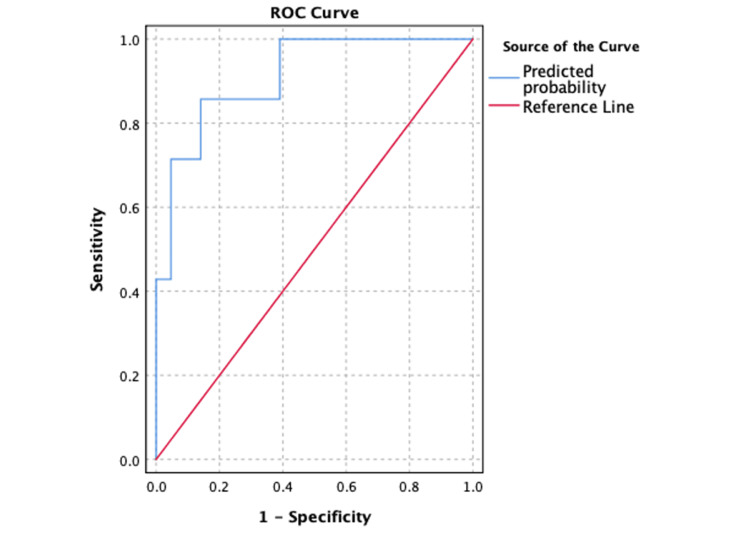
Predictive model ROC curve The model AUC = 0.911 (95% CI of 0.806 to 1.000) (SE = .054, p < .001). The Youden’s index (J_max_) is .716, corresponding to a predicted probability of 0.171 (SE = 85.7%, Specificity = 85.9%, PPV = 40.0%, NPV = 98.2%). ROC: Receiver operating characteristic; AUC: Area under the curve; SE: Sensitivity; PPV: Positive predictive value; NPV: Negative predictive value

The predicted probabilities were calculated and an ROC curve (Figure 1) was created showing an AUC of 0.911 (95% CI of 0.806 to 1.000) (SE = .054, p < .001). The model had a predicted probability of 0.81, indicating good model quality. The optimum Youden’s index (J) was calculated to be .716, corresponding to a predicted probability of 0.171 (SE = 85.7%, Specificity = 85.9%).

## Discussion

To the best of our knowledge, there has been no predictive model for CSDH recurrence in the Philippines. The overall recurrence rate of the cohort was 9.9% which is congruent with existing literature.

CSDHs are more frequently unilateral, but may be bilateral in 9.2% to 34.9% of cases [7-10]. In our study, 50 patients (70.4%) had unilateral CSDH, while 21 patients (29.6%) patients had bilateral CSDH. Studies have shown that the simultaneous bilateral drainage of CSDH lowers the risk of recurrence and need for re-treatment compared with unilateral drainage [11,12]. Other studies have not arrived at the same conclusion, citing that unilateral evacuation does not significantly influence recurrence of CSDH [13,14]. Our study has demonstrated that bilateral surgery may reduce the risk of recurrence (OR 0.005, 95% CI, p = 0.032). Most CSDH patients are elderly and have atrophic brains. Hence, doing a bilateral surgery will result to greater decompression of the intracranial space, and potentially enhance brain re-expansion.

Antithrombotic use has been consistently identified as a risk factor for the development of a CSDH. However, its role in influencing CSDH recurrence post surgery is still controversial. There have been studies that have determined that the recurrence rates are similar in patients who have no history of antithrombotic use, and studies that have showed that anticoagulants but not antiplatelets did not increase CSDH recurrence after surgery [14-16]. Our study has found a trend towards increased CSDH recurrence (OR 45.991, 95% CI, p = 0.104), though this association has not been found to be significant in our sample.

It has been hypothesized that hyperfibrinolysis plays a role in the enlargement of CSDH. Tranexamic acid is an antifibrinolytic that inhibits plasminogen activation by reversibly binding to the lysine sites on plasminogen, thereby averting the hyperfibrinolytic process and potentially preventing the progression of CSDH. Our study has found that the administration of tranexamic acid intraoperatively has led to a decrease in CSDH recurrence (OR 0.033, 95% CI, p = 0.042). Retrospective studies have proposed that tranexamic acid may be pivotal in the non-surgical management and prevention of recurrence of CSDH [17,18]. At present, randomized controlled trials are ongoing to investigate the role of tranexamic acid in conservatizing CSDH [19,20].

The length of hospital stay may be considered an aggregate measurement of overall surgical care. Our study found that prolonged hospital stay may increase the risk of CSDH recurrence once post-operative complications set in (OR 1.177, 95% CI, p = 0.032). Corollary to this, decreased hospital stay may influence recurrence by indirectly decreasing in-hospital post-operative complications. Hence, strategies like early ambulation and meticulous daily wound care have been employed. Early ambulation has been found to mitigate the risk of pneumonia and urinary tract infection with no significant increase in risk of CSDH recurrence [21].

A closed drainage system is a standard in the surgical management of CSDH. The present study has identified placement in the subdural space to decrease the risk of CSDH compared to placement in the subgaleal space (OR 0.036, 95% CI, p = 0.084). However, multiple studies have demonstrated that drain placement is associated with a reduced risk of CSDH recurrence regardless of the drain location [22-24].

**Figure 2:**
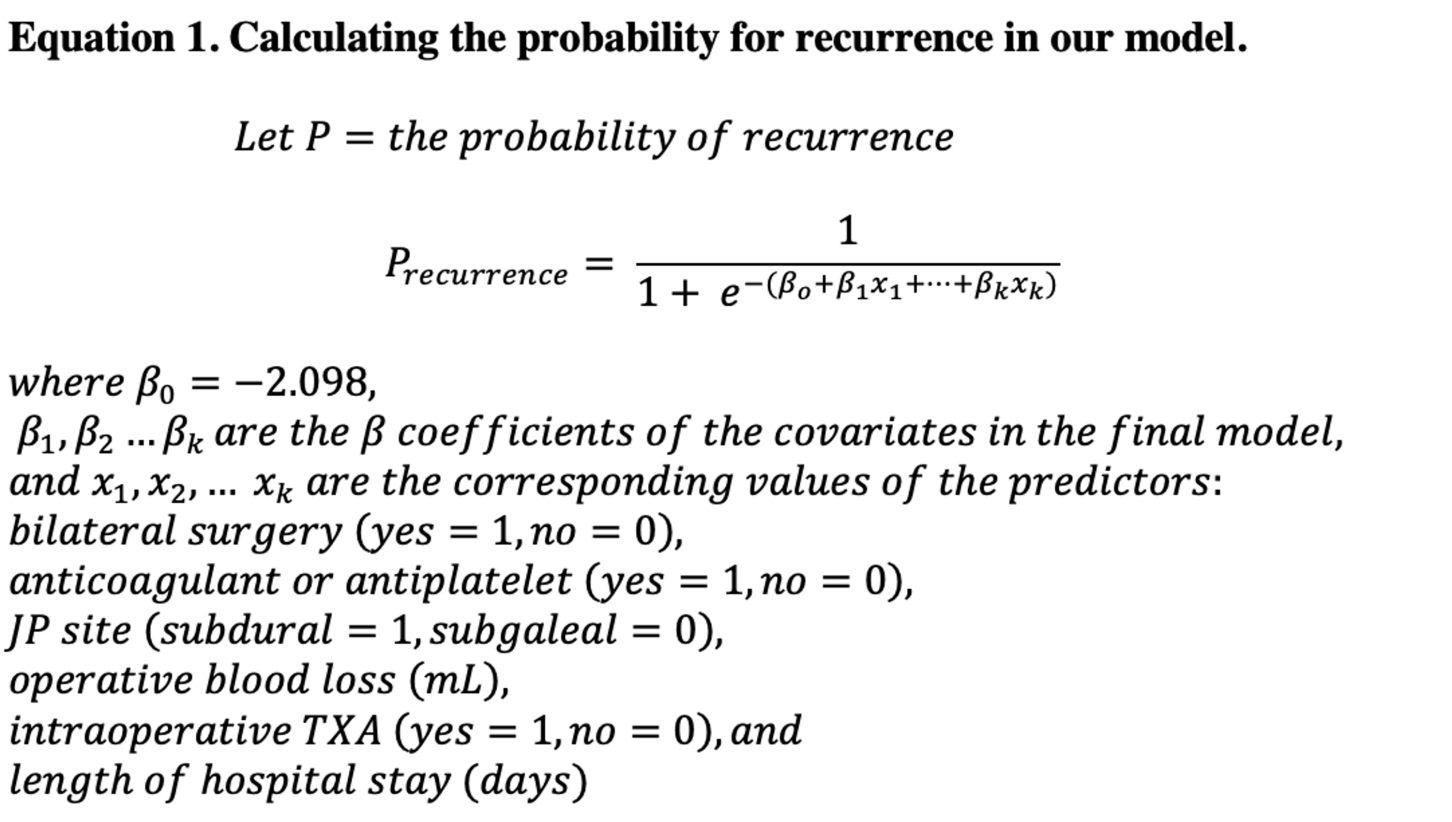
Calculating the probability for recurrence in our model

The Youden’s J_max_ coincided with a predicted probability of 0.171. If the calculation using the logistic regression equation is greater than this value, then we classify the patient as having a greater chance of CSDH recurrence (SE = 85.7%, Specificity 85.9%, PPV = 40.0%, NPV = 98.2%). The predictive model (Table 5) and equation (Figure 2) can be used in prognosticating the risk of CSDH recurrence within three months for a surgically managed patient in our institution. The authors suggest that if the predicted probability is greater than 0.171, then clinicians at our institution should follow-up a patient closely for recurrence. This will influence the post-operative care, length and interval of follow-up and avoidance of recurrence in our patients.

**Table 5:** Final model for prediction of CSDH recurrence CSDH: Chronic subdural hematoma; SE: ; OR: Odds ratio; CI: Confidence interval; JP: Jackson-Pratt drain

	β	SE	Wald χ²	df	OR	95% CI for OR	p-value
Anticoagulant or antiplatelet	3.828	2.356	2.641	1	45.991	0.454, 4654.743	.104
Bilateral surgery							
Bilateral	-2.897	1.35	4.608	1	0.055	0.004, 0.777	.032
Unilateral	–	–	–	–	1	–	–
Anticoagulant or antiplatelet	3.828	2.356	2.641	1	45.991	0.454	.104
JP site							
Subdural	-3.333	1.927	2.99	1	0.036	0.001, 1.560	.084
Subgaleal	–	–	–	–	1	–	–
Operative blood loss (mL)	-0.02	0.017	1.39	1	0.98	0.911, 1.008	.238
Intraoperative tranexamic acid							
Present	-3.425	1.682	4.147	1	0.033	0.002, 0.870	.042
Absent	–	–	–	–	1	–	–
Length of hospital stay (days)	0.163	0.076	4.6	1	1.177	1.013, 1.410	.032
Constant	-2.098	1.999	1.101	1	0.123		.294
Model Wald χ² (6, N = 71) = 18.427 (p = .005)							
-2 log likelihood = 27.293							
Cox & Snell (Nagelkerke) R^2^ = .229 (.481)							
Hosmer and Lemeshow c^2^ (8, N = 71) = 6.914, p = .546)							

Limitations and future directions

The accuracy of variables measured and small sample size limits generalizability of our model. The retrospective design of this study delimits proof of causation of the predictors to the outcome of recurrence. The predictive model the authors have created is still for validation with a prospective study with a larger cohort.

## Conclusions

The authors have created a preliminary prognostic model of subdural recurrence for our institution. We recommend validation through a prospective study with a larger cohort to improve clinical utility in the Filipino setting. We have found no significant association found between the risk of CSDH recurrence and antithrombotic use in this cohort. Decreased hospital stay may influence recurrence by indirectly decreasing in-hospital post-operative complications.

## Appendices

Appendix 1

**Table 6:** Patients with documented CSDH recurrence (N = 7) CSDH: Chronic subdural hematoma; JP: Jackson-Pratt drain

Patient	Surgery Done	Management	Remarks	Final outcome
RT, 93M	Craniectomy (Bilateral), with Drainage of CSDH (Bilateral), and Insertion of Subdural Drain (Left)	Craniotomy Right, Evacuation of Acute Subdural Hematoma, Endoscopy, Subdural Drain Insertion		Improved, Discharged
JL, 71M	Bilateral Parietal Burr, Evacuation of CSDH	Conservative	Radiologic recurrence only; no medications started	Improved, Discharged
EB, 81F	Bilateral Parietal Craniotomy, Evacuation of Subdural Hematoma	Conservative		Improved, Discharged
CA, 46F	Bilateral Burr-Hole Craniotomy with Evacuation of Subdural Hematoma	Burr-Hole Craniectomy with Evacuation of CSDH, Left		Improved, Discharged
GC, 74M	Left Frontal Craniotomy, Evacuation of Hematoma	Repeat Craniotomy, Evacuation of Subdural Hematoma Recurrence		Improved, Discharged
AR, 80M	Bifrontal Burr-Hole, Evacuation of CSDH, Insertion of JP	Right Frontal Craniotomy, Evacuation of CSDH, Insertion of JP		Improved, Discharged
CM, 81M	Right Frontal and Parietal Burr Craniectomy, Evacuation of Hematoma	Conservative	Tranexamic acid 500mg q8 x 7d; Tranexamic acid 500 mg BID x 7d; Tranexamic acid 500mg OD until Day 100	Discharged, Improved on Follow-Up
